# Methodological Confounders in Evaluating Intra‐Class Therapeutic Interchange to Evogliptin: A Closer Look at the REVISE Sub‐Analysis

**DOI:** 10.1002/edm2.70283

**Published:** 2026-07-14

**Authors:** Khushbakht Sikandar

**Affiliations:** ^1^ Nowshera Medical College Khyber Medical University Nowshera Pakistan

To the Editor,

I read with great interest the recent study by Lee et al. [1] evaluating the clinical utility of an intra‐class therapeutic interchange (ICTI) to evogliptin in patients with poorly managed type 2 diabetes mellitus (T2DM). Using a prospective, multi‐center dataset from the REVISE study, the authors demonstrate an average 0.5% reduction in HbA1c at 24 weeks, suggesting that evogliptin's distinct biochemical binding properties can successfully rescue prior dipeptidyl peptidase‐4 (DPP‐4) inhibitor treatment failures. While these real‐world insights are highly valuable for clinical practice, several unaddressed methodological and statistical confounders inherently qualify the causality attributed to the drug molecule itself.

First, the single‐arm, uncontrolled nature of the sub‐analysis leaves the findings highly vulnerable to regression to the mean (RTM). Because the inclusion criteria selectively captured patients with an elevated baseline glycemic index (HbA1c ≥ 7.0%), random biological variation over a 24‐week longitudinal follow‐up naturally biases subsequent measurements downward toward the population average. Without a parallel comparator group remaining on baseline therapy, decoupling true pharmacological efficacy from RTM artefacts is statistically impossible, as heavily documented in large‐scale glycemic screenings [2].

Second, the paper equates clinical optimization with molecular superiority, bypassing the profound impact of behavioural intervention. Real‐world treatment failure in multi‐drug oral regimens is overwhelmingly driven by covert, partial medication non‐adherence rather than biochemical drug resistance [3]. The physical execution of an intra‐class switch acts as a powerful clinical “reset,” intensifying physician engagement and temporarily spiking patient adherence. Because baseline compliance was not objectively measured via blood plasma drug concentrations, the observed 0.5% HbA1c drop is likely a reflection of renewed adherence rather than evogliptin's structural binding affinity.

Finally, the baseline background therapy across the prior DPP‐4 inhibitor subgroups was highly heterogeneous. According to Table 1, the baseline metformin doses varied severely, ranging from 735.1±308.9mg in the saxagliptin arm to 1137.5±410.3mg in the anagliptin arm. Such stark disparities in background therapeutic intensity introduce substantial confounding variables that compromise the predictability of the multivariable linear regression models used to isolate individual switch outcomes [4].

In conclusion, while an intra‐class switch to evogliptin serves as a pragmatic and safe option to break clinical inertia, the current data structure cannot definitively prove a drug‐specific pharmacological superiority over sister compounds. To validate true chemical rescue efficacy, future investigations must deploy randomized controlled trials featuring a continuation arm as an active baseline control.

## Author Contributions

**Khushbakht Sikandar:** conceptualization, investigation, formal analysis, writing – review and editing, writing – original draft.

## Funding

The author has nothing to report.

## Ethics Statement

The author has nothing to report.

## Conflicts of Interest

The author declares no conflicts of interest.

## Linked Article

This article is linked to http://dx/doi.org/10.1002/edm2.70270.

## Data Availability

Data sharing not applicable to this article as no datasets were generated or analysed during the current study.
